# Multinational Cholera Outbreak after Wedding in the Dominican Republic

**DOI:** 10.3201/eid1711.111263

**Published:** 2011-11

**Authors:** Mercedes Laura Jiménez, Andria Apostolou, Alba Jazmin Palmera Suarez, Luis Meyer, Salvador Hiciano, Anna Newton, Oliver Morgan, Cecilia Then, Raquel Pimentel

**Affiliations:** Author affiliations: Field Epidemiology Training Program, Santo Domingo, Dominican Republic (M.L. Jiménez,);; Ministry of Public Health, Santo Domingo (M.L. Jiménez, L. Meyer, S. Hiciano, C. Then, R. Pimentel);; New Jersey Department of Health and Senior Services, Trenton, New Jersey, USA (A. Apostolou);; Centers for Disease Control and Prevention, Atlanta, Georgia, USA (A. Apostolou, A. Newton);; Pan American Health Organization, Santo Domingo (A.J. Palmera Suarez);; Atlanta Research and Education Foundation, Decatur, Georgia, USA (A. Newton);; Centers for Disease Control and Prevention, Santo Domingo (O. Morgan)

**Keywords:** cholera, Vibrio cholerae, outbreak, serotype Ogawa, Dominican Republic, bacteria

## Abstract

We conducted a case–control study of a cholera outbreak after a wedding in the Dominican Republic, January 22, 2011. Ill persons were more likely to report having consumed shrimp on ice (odds ratio 8.50) and ice cubes in beverages (odds ratio 3.62). Travelers to cholera-affected areas should avoid consuming uncooked seafood and untreated water.

Over the past century, no cholera cases had been reported in the Dominican Republic. The first cholera cases were reported on November 15, 2010, associated with the epidemic in Haiti. From November 15, 2010, through January 22, 2011, a total of 1,115 suspected cases and 280 laboratory-confirmed cases were reported by the Dominican Republic Ministry of Health (*1*).

On January 25, 2011, the Dominican Republic Ministry of Health was notified that 5 Venezuelan nationals had been hospitalized with cholera-like illness. All patients had attended a large wedding reception at a luxury tourist resort in La Romana Province, Dominican Republic, on January 22. At the reception were 216 local workers and 540 guests; ≈90% were residents of Venezuela, and the others were from the Dominican Republic, Mexico, the United States, and Spain. In La Romana Province, no other cases had previously been identified, and during the week of the outbreak, all reported cases were associated with the wedding.

## The Study

We conducted a case–control study to identify foods and beverages consumed at the wedding banquet that were associated with diarrheal illness. A questionnaire developed by Dominican Republic health authorities, which addressed only food items consumed at the banquet, was sent to national health authorities in the 4 countries where guests originated. They were asked to administer it to wedding attendees and return completed questionnaires to the Dominican Republic for analysis.

Case-patients were identified from routine surveillance reports in the Dominican Republic and the United States. We also actively sought case-patients by asking the venue personnel, event organizer, and other case-patients to inform us about other persons at the banquet from the same country of origin. A patient with a suspected case was defined as a person who had watery diarrhea during January 22–28, 2011, and had consumed food and beverages at the banquet. In addition to meeting suspected-case criteria, a patient with a confirmed case also had laboratory confirmation of infection by culture for *Vibrio cholerae* O1 or agglutination for serotype Ogawa. Controls were identified by event organizers or other banquet attendees as guests or workers at the banquet who consumed food and beverages but did not have diarrhea during January 22–28, 2011. We intended to recruit at least twice as many controls as case-patients. We calculated odds ratios (ORs) and 95% confidence intervals (CIs) regarding whether consuming particular foods and beverages was associated with being a case-patient (with suspected and confirmed cases) and controls. Food and beverage items potentially associated with illness in a univariable analysis (p<0.10) were included as independent dichotomous predictor variables in a multivariable logistic regression model with case status as the dependent dichotomous variable. We used StataCorp 2005 Release 9 (College Station, TX, USA) for the analysis. A Ministry of Health environmental inspector interviewed the event caterers about purchase, transport, storage, preparation, and service of food and beverages at the banquet.

We identified 42 case-patients (25 with confirmed and 17 with suspected cases) and 62 controls; all agreed to participate in the study. Questionnaires were completed between January 24 and March 3, 2011. Twenty-two (51%) case-patients and 59 (97%) controls were from the Dominican Republic (Table 1). Of case-patients, 24 (57%) were wedding guests, 16 (38%) were workers, and 2 (5%) did not attend the reception but ate leftover food from the banquet. Of 62 controls, only 6 (10%) were guests and 56 (90%) were workers (Table 1). Only data from Venezuelan case-patients who received a diagnosis in the Dominican Republic were available for analysis. We did not specify how case-patients were identified.

**Table 1:** Characteristics of case-patients and controls associated with a cholera outbreak after a wedding, Dominican Republic, January 2011

Characteristic	No. (%) case-patients, n = 42	No. (%) controls, n = 62	p value*
Sex			
M	33 (77)	54 (89)	0.18
F	9 (23)	8 (12)	
Age group, y			
15–24	8 (23)	9 (15)	0.07
25–34	8 (19)	20 (33)	
35–44	8 (16)	18 (28)	
45–54	6 (14)	9 (15)	
55–64	9 (21)	3 (5)	
>65	3 (7)	3 (5)	
Role			
Guest	24 (58)	6 (8)	<0.001
Server or food handler	9 (21)	22 (36)	
Worker	9 (21)	34 (56)	
Country of origin			
Spain	1 (2)	1 (2)	<0.001
United States	10 (26)	2 (2)	
Dominican Republic	22 (51)	59 (97)	
Venezuela†	9 (21)	0	

*p value based on χ^2^ test for each contingency table. †Case-patients were identified and interviewed in the Dominican Republic.

Median age of case-patients was 42.5 years (range 16–84 years); 33 (79%) were male. All experienced watery diarrhea, accompanied by dehydration (28 patients [67%]), nausea (13 [30%]), vomiting (15 [36%]), or cramps (8 [19%]). Time to illness onset was 10 hours to 6 days (Figure). Thirty-four (81%) case-patients were given antimicrobial drugs, and 22 (52%) were hospitalized; all recovered.

**Figure:**
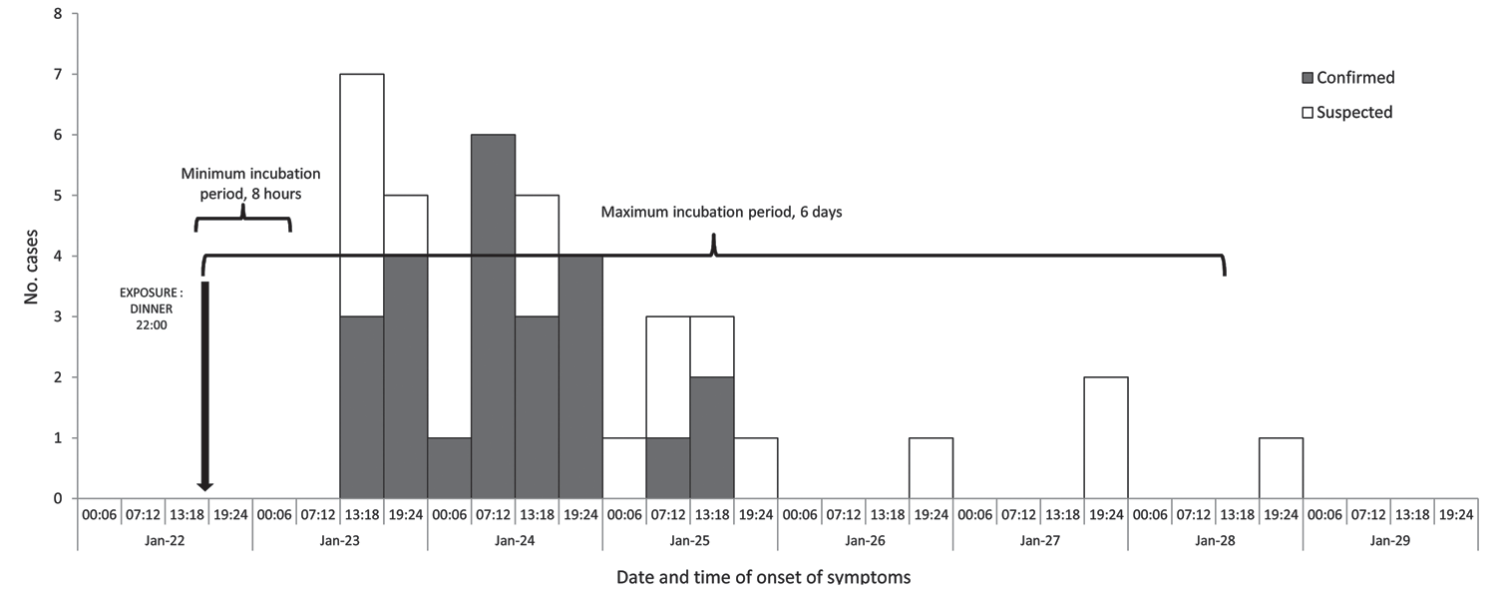
Date and time of onset of cholera cases (N = 40) associated with wedding in La Romana, Dominican Republic, January 2011 (2 case-patients not represented here because time of symptom onset was not reported).

Odds were higher for case-patients than controls for having consumed cooked shrimp on ice (OR 8.5, 95% CI 3.3–21.71), cooked langostinos (squat lobster) (OR 2.9, 95% CI 1.1–7.7), and beverages with ice cubes (OR 3.6, 95% CI 1.4–9.3), but odds were lower for having consumed mixed rice (OR 0.1, 95% CI 0.01–0.9) or other foods (OR 0.13, 95% CI 0.05–1.03) (Table A1). When these variables were included in a multivariable logistic regression, cooked shrimp on ice (OR 10.8, 95% CI 3.3–35.4), ice cubes (OR 4.1, 95% CI 1.3–13.2), and mixed rice (OR 0.04, 95% CI 0.003–0.5) remained significantly associated with case status (Table 2).

**Table 2:** Odds of consuming specific food and beverage items during a wedding, Dominican Republic, January 2011*

Food item consumed	No. (%) case-patients, n = 42	No. (%) controls, n = 62	OR (95% CI)	p value
Cooked shrimp on ice	25 (60)	9 (15)	10.82 (3.31–35.35)	<0.001
Langostinos	14 (33)	9 (15)	2.23 (0.56–8.81)	0.254
Mixed rice and vegetables	1 (2)	12 (19)	0.04 (0.003–0.45)	0.009
Other food	1 (2)	10 (16)	0.18 (0.02–1.74)	0.139
Ice cubes in beverages	16 (38)	9 (15)	4.10 (1.28–13.16)	0.018

*OR, odds ratio; CI, confidence interval. Pearson goodness-of-fit for the regression model <0.001 (10 df, χ^2^ 38.19). Because exponentiated coefficients from the logistic regression model are shown in the table, the regression intercept (log odds for non–case-patients) is not shown.

Environmental inspection found that the food was prepared by a caterer outside of the resort. Shrimp and lobster came from the Dominican Republic (Higuey in La Altagracia Province), and the langostinos came from Beata Island in Perdenales Province and Anse-A-Pitre in southeastern Haiti. No samples were available for testing. Inspection of the caterer’s kitchen revealed poor food-handling practices, including improper refrigeration, poor hand hygiene, and nonchlorinated water supply. On the night of the wedding reception, food was set out at 7:00 pm but not consumed until 10:00 pm. Shrimp and langostinos were served on ice or ice sculptures.

## Conclusions

After a wedding banquet in La Romana Province, Dominican Republic, many persons with gastrointestinal symptoms had laboratory evidence of infection with *V. cholerae* O1 Ogawa, the cholera serotype identified in the ongoing outbreak on Hispaniola. Cases were positively associated with consumption of cooked shrimp on ice and ice cubes. However, case-patients were not more likely to have eaten shrimp in vinaigrette or shrimp kebab, which may indicate contamination during serving on ice. The treatment of shrimp in vinaigrette with acid (vinegar) and shrimp kebabs with heat may have killed *V. cholerae* that was present before preparation. Seafood has long been established as a vehicle for cholera transmission (*2**–**5*).

Our investigation had several limitations. We were unable to access information from 90% of wedding guests from Venezuela, potentially introducing representation bias. We did not collect information about other meals served to guests and workers before and after the wedding banquet. We did not have itineraries of guests and could not assess whether they had traveled to other parts of Hispaniola affected by cholera before the wedding. We were unable to recruit 2 controls per case-patient, thus reducing the study’s statistical power to detect any weak associations. The international nature of the investigation made it difficult to obtain information in a timely manner, potentially increasing recall bias from widespread media reports of seafood being implicated in the outbreak. Most controls were workers from the Dominican Republic, and most case-patients were guests from Venezuela, which may have led to differential food and drink preferences or differential access to food items on the menu. Moreover, nearly one fifth of case-patients were food handlers, and we cannot exclude the possibility that they cross-contaminated the food or items served. Food and drink served at the reception were unavailable for testing, and we had no information about the source of water used to make ice cubes.

Recommendations to establish a cold chain, use chlorinated water, and exclude ill food handlers were provided to the catering facility and seafood provider. After the investigation, prevention and control measures, including closer adherence to the existing prohibition of importation of high-risk food items from Haiti, were implemented in tourist hotels across the Dominican Republic.

This report highlights the need for international collaboration between public health entities during cholera epidemics. Cholera prevention materials that include information on high-risk food items, such as shellfish and ice should be provided to travelers before they visit potentially cholera-affected areas. Increased awareness and active disease surveillance can help control the spread of cholera outbreaks and prevent secondary transmission.

## Appendix

**Table A1:** Association of consumption of specific foods and beverages and cholera case status, Dominican Republic, January 2011*†

Food item consumed	No. (%) case-patients ate, n = 42	No. (%) controls ate, n = 62	OR (95% CI)	p value
Cooked lobster	18 (43)	21 (34)	1.46 (0.65–3.28)	0.354
Cooked shrimp on ice	25 (60)	9† (15)	8.50 (3.32–21.71)	<0.001
Shrimp in vinaigrette	7 (17)	0	NA	NA
Shrimp kebab	14 (33)	12 (19)	2.08 (0.85–5.12)	0.110
Langostinos	14 (33)	9 (15)	2.94 (1.13–7.65)	0.027
Cooked langostinos in creamy sauce	9 (21)	10 (16)	1.42 (0.52–3.86)	0.494
Sushi	6 (14)	6 (10)	1.56 (0.47–5.20)	0.473
Seviche	8 (19)	6 (10)	2.20 (0.70–6.87)	0.177
Smoked salmon	4 (10)	5 (8)	1.20 (0.30–4.76)	0.795
Caviar	2 (5)	0	NA	NA
Beef empanada	6 (14)	8 (13)	1.13 (0.36–3.52)	0.839
Crab empanada	2 (5)	6 (10)	0.47 (0.09–2.43)	0.366
Pork rib	7 (17)	12 (19)	0.83 (0.30–2.33)	0.728
Serrano ham	5 (12)	5 (8)	1.54 (0.42–5.69)	0.517
Hamburger	3 (7)	9 (14)	0.52 (0.13–2.08)	0.355
Ham sandwich	1 (2)	5 (8)	0.40 (0.03–2.47)	0.251
Tuna salad sandwich	0	3 (5)	NA	NA
Pate	0	6 (10)	NA	NA
Vinaigrette	7 (17)	8† (13)	1.33 (0.44–3.98)	0.616
Green beans	1 (2)	7 (11)	0.19 (0.02–1.62)	0.129
Grilled vegetables	2 (5)	7 (11)	0.39 (0.08–1.99)	0.259
Mixed rice and vegetables	1 (2)	12 (19)	0.10 (0.01–0.81)	0.031
Fries	5 (12)	11 (18)	0.63 (0.20–1.96)	0.421
Soda	3 (7)	0†	NA	NA
Whisky	3 (7)	2† (3)	2.27 (0.36–14.20)	0.381
Water	11 (26)	10† (16)	1.81 (0.69–4.75)	0.229
Ice cubes in beverages	16 (38)	9 (15)	3.62 (1.41–9.29)	0.007
Whisky with ice	2 (5)	0	NA	NA
Water with ice	1 (2)	1 (2)	1.49 (0.09–24.46)	0.781
Other food	1 (2)	10 (16)	0.13 (0.05–1.03)	0.053
Other drink	11 (26)	12 (19)	1.48 (0.58–3.75)	0.411

*OR, odds ratio; CI, confidence interval; NA, not available.

†N = 61.
